# Detecting dynamic spatial correlation patterns with generalized wavelet coherence and non-stationary surrogate data

**DOI:** 10.1038/s41598-019-43571-2

**Published:** 2019-05-14

**Authors:** Mario Chavez, Bernard Cazelles

**Affiliations:** 1 CNRS UMR-7225, Hôpital de la Pitié-Salpêtrière, 75013 Paris, France; 2 IRD-UPMC UMI-209, UMMISCO, 93143 Bondy, France; 3 CNRS UMR-8197, IBENS, Ecole Normale Supérieure, 75005 Paris, France

**Keywords:** Statistical methods, Statistics, Nonlinear phenomena

## Abstract

Time series measured from real-world systems are generally noisy, complex and display statistical properties that evolve continuously over time. Here, we present a method that combines wavelet analysis and non-stationary surrogates to detect short-lived spatial coherent patterns from multivariate time-series. In contrast with standard methods, the surrogate data proposed here are realisations of a non-stationary stochastic process, preserving both the amplitude and time-frequency distributions of original data. We evaluate this framework on synthetic and real-world time series, and we show that it can provide useful insights into the time-resolved structure of spatially extended systems.

## Introduction

Synchronization is a fundamental phenomenon described in many biological and physical contexts for which there are two or more interacting oscillatory systems^1^. The interactions between coupled oscillators in real systems continuously create and destroy synchronised states, which can be observed as noisy and transient coherent patterns. The statistical detection of spatial synchrony in networks of coupled dynamical systems is therefore of great interest in disciplines such as geophysics, physiology and ecology^2–5^. Coherence is generally defined as the correlation between concurrent time series of a variable measured from several processes, whereas synchrony is referred to as the degree to which their fluctuations behave similarly over time^1^. At various points throughout this paper, the terms synchrony and coherence are interchangeably used to describe the degree to which different process evolve in a similar way.

Statistical significance of transient coherent patterns cannot be assessed by classical spectral measures and tests, which require signals to be stationary^4–6^. Synchrony estimators based on nonparametric methods have the advantage of not requiring any assumption on the time-scale structure of the observed signals. Among them, measures of synchrony or coherence based on wavelet transforms have been widely used to detect interactions between oscillatory components in different real systems, i. e. neural oscillations, business cycles, climate variations or epidemics dynamics^2–5^.

In recent years, different significance tests for the wavelet cross-spectrum or wavelet coherence have been developed to detect oscillatory patterns with covarying dynamics^4–10^. Unfortunately, the statistical assumptions of these tests are not always compatible with the structure of the data considered, and significance levels often depend on the structure of the wavelets applied^7–10^. A rigorous theoretical framework cannot therefore be derived, and Monte Carlo simulations have to be performed to estimate the significance level^7–10^.

Surrogate data techniques have been proposed as non-parametric resampling methods for testing general hypotheses on data without making assumptions on the underlying generating process^11–13^. However, time series measurements from real systems generally display irregular fluctuations, long-term trends, or a time-varying spectra. Such properties are incompatible with the main assumptions of standard surrogate data based on Fourier transform^14–18^. Indeed, time-varying spectral properties (or any relevant description of these characteristics) are generally indicators of non-stationarity^19^, or non-autonomous dynamics^20^.

Recently, parametric models have been also applied to test wider classes of null hypothesis, including non-stationary behaviour^17,18^. Some limitations of these approaches include the relatively large basis dimension needed to obtain good optimisation, and the monitoring needed to control the instabilities in the estimated model^21,22^. When signals are available from a large number of trials/subjects, non-stationary surrogates can be obtained by simply shuffling the time series between trials/subjects^12^. Nevertheless, this method assumes similar time-varying spectral properties across trials/subjects. Recent studies have proposed the use of discrete wavelet transforms (DWT) for resampling time series such that the multiscale structure of original data is preserved^23–26^. The main advantage of DWT is their ability to concentrate the signal’s variance in a limited number of coefficients. Nevertheless, the number of data points heavily influences this decomposition (the number of scales); which may render the scale decompositions difficult to interpret^4,5^. Although continuous wavelets often yield a redundant decomposition across scales, they are more robust to noise as compared with other decomposition schemes^4–6,27^.

In this work, we use a continuous wavelet-based approach to detect spatial coherent patterns in non-stationary multivariate observations. We generalise the wavelet coherence to multivariate time series and we extend the classic phase-randomised surrogate data algorithm to the time-frequency domain for generating non-stationary surrogates. This procedure preserves both the original amplitude and time-frequency energy (spectrogram) distributions. We assess the reliability and performance of our method by comparing our results with those obtained from classical stationary surrogate method and a non-stationary surrogate algorithm based on discrete wavelet transform (DWT). Compared with other surrogate algorithms, our method better replicate the time-frequency structure of real data. Non-stationary surrogates are used to assess the significance of transient coherent patterns found in multivariate time series. We evaluate the proposed method in different synthetic and real-world non-stationary data, and we show that this approach can substantially improve the detection of time-varying spatial coherence.

## Results

### Replication of the time-frequency structure by surrogate data

**Figure 1:**
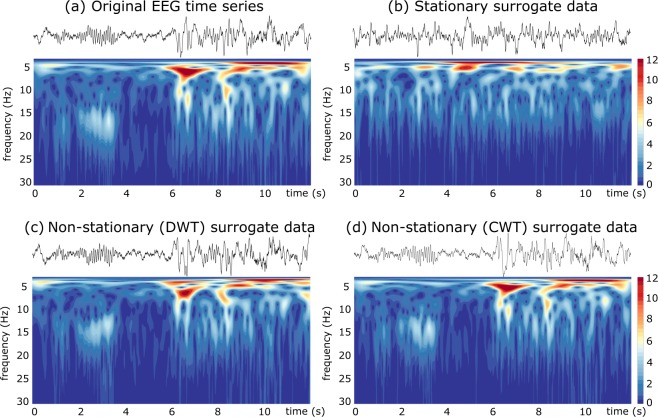
Exemple of the time-frequency (TF) structure for different surrogate algorithms applied to epileptic EEG data. (**a**) The original time series after reconstruction by the wavelet filtering, (**b**) surrogate data generated with the iterative Amplitude Adjusted Fourier Transform (iAAFT) algorithm, (**c**) non-stationary surrogate data test with a DWT (the iAAWT algorithm) and, (**d**) surrogate generated with our algorithm. The color maps code for $|({W}_{x}(t,f))|$ values. Wavelet analysis of EEG were done over the frequency range 0–128 Hz (with a frequency resolution of 0.1 Hz). For better visualisation, spectra are displayed only for $f < 30\,{\rm{Hz}}$.

To illustrate our surrogate data method, we consider an electroencephalographic (EEG) recording from a pediatric subject with intractable seizures^28,29^. The non-stationarity of epileptic EEG signals is clearly illustrated in Fig. 1a. One can notice that the frequency content of epileptic oscillations may change rapidly across time over a range of frequencies. The time-frequency plot exhibits a short fast oscillatory behavior (15–20 Hz) around *t* = 3 sec followed by slow and large oscillations accompanying the epileptic seizure after *t* = 6 sec. As depicted in Fig. 1b, classical stationary surrogate data (here we used the iAAFT algorithm^11,13^) is not able to replicate the non-stationary oscillations embedded in the original signal. Compared with standard surrogate method, DWT-based surrogates replicate better the TF structure of original data (Fig. 1c). Nevertheless, our algorithm is able to conserve the time-varying spectrum of original signal, as illustrated in Fig. 1d (refer to Supplementary Information for additional details and comparisons.). Distributions in Fig. 1e confirm that the three surrogate algorithms conserve the amplitude distributions.

**Figure 2:**
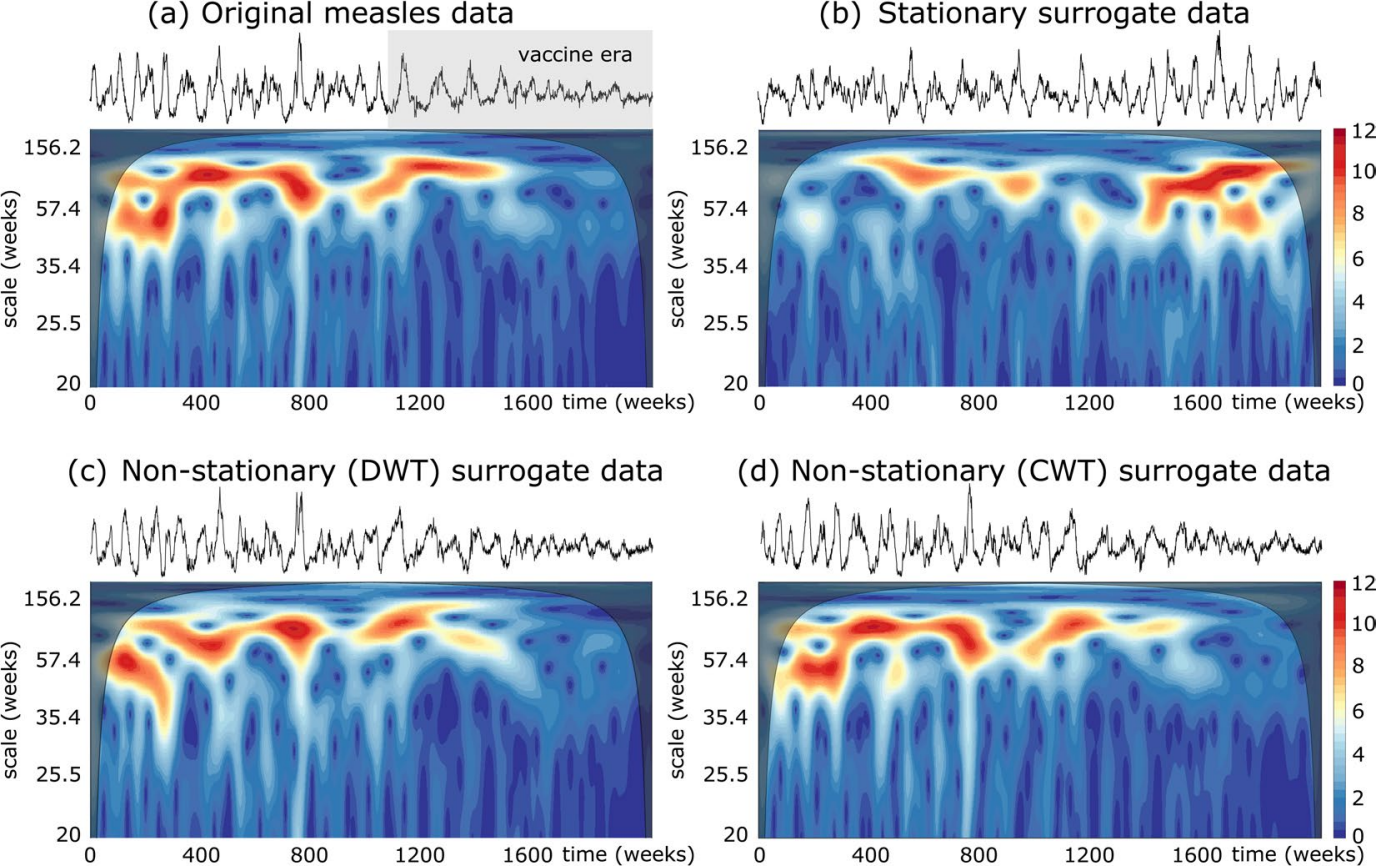
Exemple of the TF structure for different surrogate algorithms applied to the squared root transformed measles data. Same stipulations as in the caption of Fig. 1. Wavelet analysis of measles data were done over the scale range 2–1000 weeks, spanned over 2496 scales. For better visualisation, spectra are displayed only scales larger than 20 weeks. Gray box in upper plot a indicates the vaccine era. Black transparent maps of time-frequency plots indicate the cone of influence that delimits the regions not influenced by edge effects (As the wavelet is centered close to the edges of time series, edge effects occur. The area of the TF plane where such effects are relevant, the so-called cone of influence, was chosen as the *e*-folding time of the Morlet wavelet function^6^).

Another paradigmatic example of non-stationary spatial synchrony is that observed in population dynamics. Here, we consider the weekly measles case notifications in Liverpool, UK^30,31^. Measles epidemics generally exhibit a non-stationary dynamics with a regular and highly epidemics before nationwide vaccination programs, and an irregular and spatially uncorrelated dynamics in the vaccine era. As illustrated in Fig. 2, the data display multiannual cycles that dramatically varies with time, specially the annual component, which is clearly attenuated after vaccination. This rich behavior can not be encompassed by classical stationary surrogate data (Fig. 2b). DWT-based surrogates replicate better the TF structure of original data (Fig. 2c). Nevertheless, our method based on continuous wavelet transform perfectly keeps the variations of epidemic periods observed in the original time series (Fig. 2d). Plot in Fig. 2e confirms that the three surrogate algorithms conserve the amplitude distributions.

### Detecting global synchronization in synthetic data

**Figure 3:**
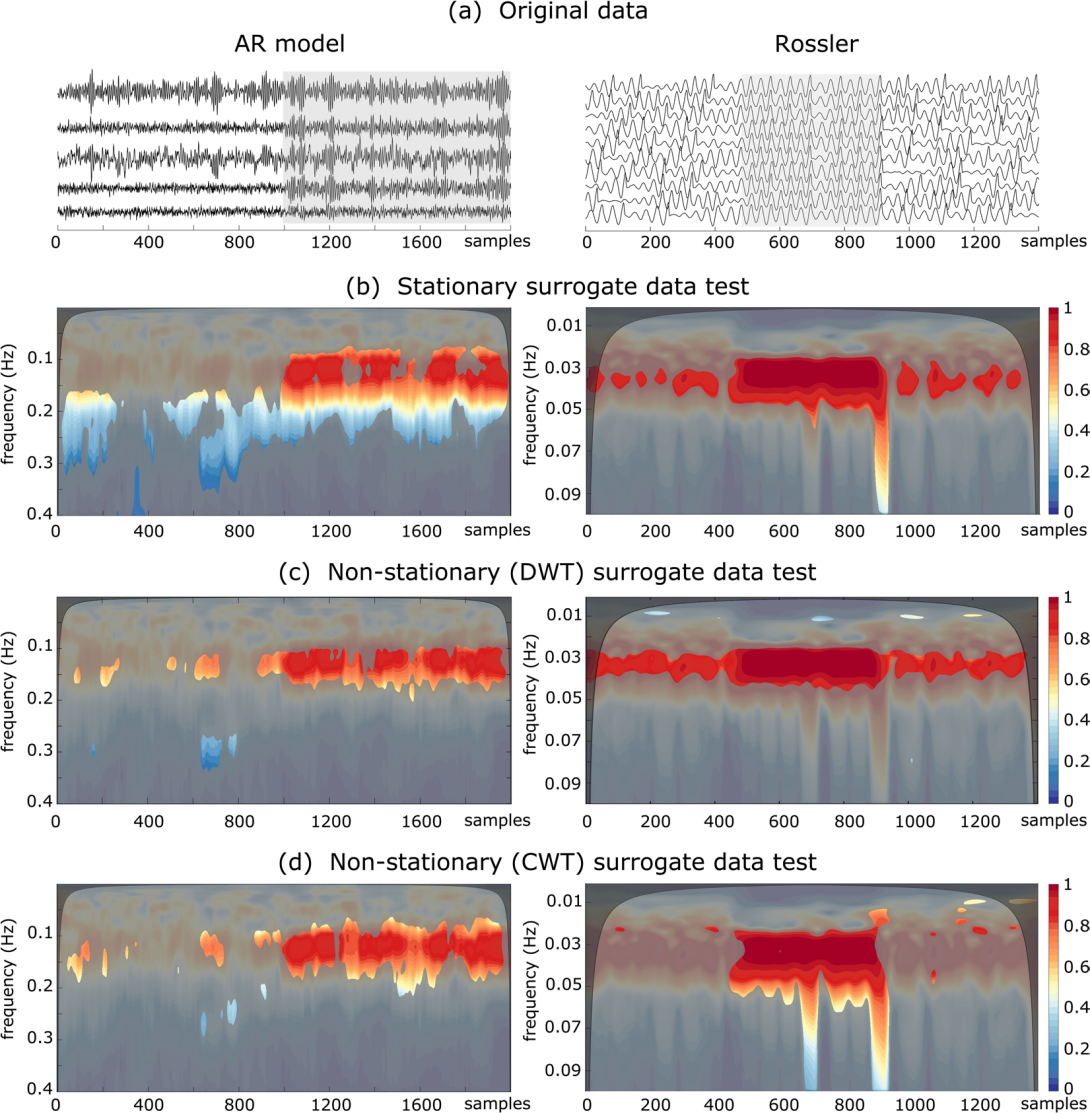
${\rm{\Psi }}(t,f)$ values estimated from synthetic time series and statistical differences with those values obtained from different surrogate data. (**a**) The original time series (gray boxes delimitate the region where the system is synchronized), (**b**) surrogate data test based on the iAAFT algorithm (**c**) non-stationary surrogate data test based on a DWT (the iAAWT algorithm) and (**d**) our algorithm based on a continuous wavelet transform. Wavelet analysis were done over the frequency range 0–0.5 Hz with a frequency resolution of 10^−3^ Hz (for a normalized sampling rate of 1 Hz). The color maps code for ${\rm{\Psi }}(t,f)$ values. Unmasked color regions in panels B-C indicate the significant levels. Black transparent maps indicate the cones of influence (As the wavelet is centered close to the edges of time series, edge effects occur. The area of the TF plane where such effects are relevant, the so-called cone of influence, was chosen as the *e*-folding time of the Morlet wavelet function^6^).

In Fig. 3a–d we report, respectively, the synthetic time series generated by different models, the significant coherent components detected by the varying spatial coherence, ${\rm{\Psi }}(t,f)$, in combination with classical surrogate algorithm and with other non-stationary surrogate data. Results reveal that stationary randomizations detect several large spurious synchrony patches on the time-frequency plane, e.g. the large patches before $t=1000$ for the coupled AR model, or those out of the synchronous region for the coupled Rössler model ($500 < t < 900$). This is mainly due to the oscillations created over the whole segment by the stationary surrogate algorithm. Although the DWT-based algorithm imitates the time-scale structure of original data, it still detects some spurious coherent patches. Conversely, a detection based on our method considerably reduces the number of false coherent patches, while it clearly identifies the main regions with the highest spatial coherence. Remarkably, results show that the combination of ${\rm{\Psi }}(t,f)$ with non-stationary surrogate data, constitutes a good criterion to assess spatial coherence in the case of nonlinear dynamical time series.

### Global coherence in real spatial systems

**Figure 4:**
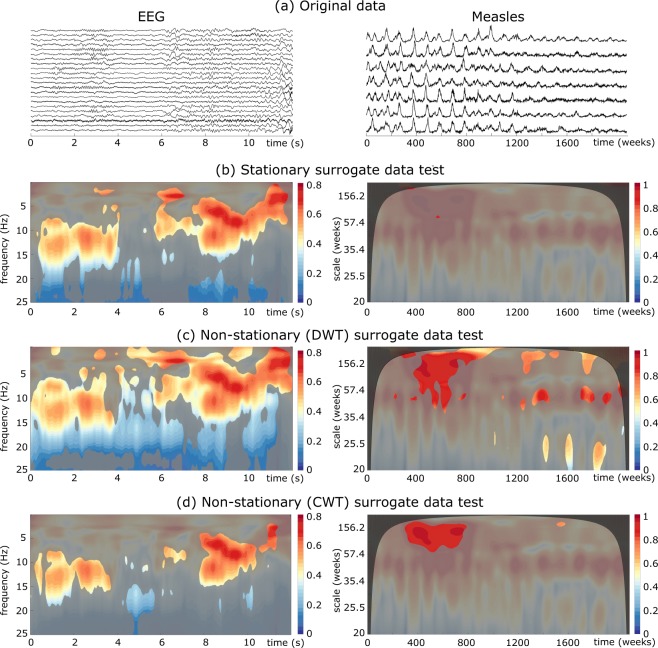
${\rm{\Psi }}(t,f)$ values estimated from real spatial systems and statistical differences with those values obtained from different surrogate data. For measles data, missing values in each original time series were imputed using a local average, i.e. the mean of the two neighboring time points. Same stipulations as in the caption of Fig. 3.

The situation with EEG data is illustrated in left panels of Fig. 4. The first crucial observation is that, as expected in epilepsy dynamics, spatial coherent patterns are not time invariant, but instead they exhibit a rich time-frequency structure during seizure evolution. Results clearly show that classical surrogate data test may yield to the detection of large synchronous regions, specially at high frequency bands ($f\geqslant 20$) Hz. Similarly, a test of significance based on the non-stationary DWT also yields to the detection of large and spurious synchronous regions, as those detected between 15–25 Hz during practically the whole signal. In contrast, our non-stationary surrogates improves the time-frequency localization of spatial correlation patterns. A first synchronous pattern seem to involve the low-amplitude fast oscillations often observed during the first seconds of epileptic seizures. Interestingly, the absence of significative values of ${\rm{\Psi }}(t,f)$ between *t* = 4 − 8 s suggest a desynchronization of some cerebral structures during the build-up of epileptic seizures, just before a wide synchronous spreading to the ensemble of the brain at *t* = 8 s. This fully agrees with previous findings suggesting a neural desynchronization before the propagation of seizures which could facilitate the development of local pathological recruitments^32,33^.

Right panels of Fig. 4 show the results for the measles data. We observe from ${\rm{\Psi }}(t,f)$ values that global interactions between major epidemics change relatively smoothly through time. Classic surrogate analysis can capture epidemic’s dynamics at different scales, but does not allow a proper description when they change with time. Indeed, standard surrogate data test reveals no significant spatial correlation patterns. The use of surrogates obtained by the DWT reveals the major biennal synchronous epidemic component characteristic of the pre- vaccine era. Nevertheless, it also detects short periods of spatial interactions between annual oscillations, often associated to spurious correlations produced by seasonal variations^34^. Conversely, our approach clearly detects the main changes in spatial correlation structure: a high spatial coherence between the major epidemic (mainly biennial) component of time series is clearly identified in the pre-vaccine era. The interactions between the smaller epidemics with longer periods observed after vaccination are not found to be statistically significant. This is a remarkable result as it supports previous findings that during the pre-vaccination era, measles dynamics is characterized by a high spatial correlation of biennal epidemic patterns, while the vaccination eliminates large epidemics yielding thus a significant spatial decorrelation^30,31^.

## Discussion

To summarize, we have addressed a fundamental problem in complex systems: detecting, from scalar observations, the time scales involved in spatial interactions of oscillators with time-varying spectral components. Classical surrogate data tests require time-series to be stationary. Nevertheless, data recorded from real-world systems are generally noisy and non-stationary. In order to study their interactions we propose a complementary approach based on wavelet analysis. Wavelet coherence is generalized as a method for detecting transient but significant coherence between multivariate nonlinear signals. The classic surrogate algorithm is also generalized to produce non-stationary surrogates. Results from several artificial and real non-stationary, linear and nonlinear time series, demonstrate the advantages of our approach.

We have assessed the performance of our surrogate method by comparing our results with those obtained from a non-stationary surrogate algorithm based on discrete wavelet transform. Results confirm that, compared with stationary Fourier-based and non-stationary DWT-based surrogate algorithms, our method replicates better the time-frequency structure of original data (see Supplementary Information for additional results). Other wavelet-based methods have been used to analyse the relationships between multivariate signals^1,35–37^. Nevertheless, standard significance tests assume stationarity of observations, which strongly affects the significance of the detected coherent patterns. Our results also provide evidence of the constructive role of non-stationary surrogate data to uncover changes of correlation patterns in multivariate time series. When a sufficient number of surrogates is applied (e.g. 100 in our examples, but see Fig. S3 in Supplementary Information), our test constitutes a good criterion to assess spatial coherence in the case of time series with time varying spectra.

The proposed estimator considers only interactions at the same oscillatory frequency, or 1:1 synchronization. A more general case of *n*:*m* synchronization should include synchrony fluctuations between two arbitrary oscillatory frequency components. Nevertheless, when the components are extracted by time-frequency approaches, arbitrary pairs (*n*, *m*) may yield unstable synchronies, with a large estimator?s variance that depends on the frequency values and the *n*/*m* ratio^38^. In the case of any multivariate method, future research should address the constraints for determining the (*n*, *m*) pair to ensure a robust multivariate *n*:*m* synchrony.

The detection of spatial correlations in other multivariate data (e.g. financial or climate time series) might provide meaningful insights into the structure of other spatially extended systems. Although the proposed method uses linear coherence to quantify the time-varying interactions, the framework could also add new functionality to current non-linear analysis techniques. The algorithm can be therefore extended any other quantifier of spatio-temporal variability (e.g. phase wavelet synchrony), provided it yields a real and symmetric matrix of interactions at different ponts in the time-frequency plane. This method could provide useful clues about the nature of the underlying processes in many biological and physical contexts.

## Methods

### Generalizing the wavelet coherence

We start by considering the time-frequency (TF) distributions obtained by convolving a time series *x*(*t*) with a scaled and translated version of a chosen mother wavelet ${w}_{s,\tau }(t)=\frac{1}{\sqrt{s}}w(\frac{t-\tau }{s})$. Throughout the paper, we consider the complex Morlet wavelet defined as $w(t,{f}_{0})=A\,\exp (\,-\,{t}^{2}/2{\sigma }_{t}^{2})\times \exp (i2\pi {f}_{0}t)$. Wavelets were normalized with $A=({\sigma }_{t}\sqrt{\pi }{)}^{-\mathrm{1/2}}$. The width of each wavelet function ($m={f}_{0}$/*σ*_*f*_) was chosen to be 5 (it makes the Morlet wavelet approximately analytic^27^), where ${\sigma }_{f}=1$/2*πσ*_*t*_.

To quantify the relationships between two non-stationary signals, *x*_*i*_(*t*) and *x*_*j*_(*t*), the wavelet cross-spectrum is given by ${W}_{i,j}(t,f)={W}_{i}(t,f){W}_{j}^{\ast }(t,f)$, where * denotes the complex conjugate operator and ${W}_{k}(t,f)$ is the wavelet transform of signal *x*_*k*_(*t*). Let us now consider *M* zero-mean time series ${x}_{1}(t),\ldots ,{x}_{M}(t)$, and define the complex coherence spectrum as ${C}_{i,j}(t,f)=\frac{\langle {W}_{i,j}(t,f)\rangle }{{\Vert \langle {W}_{i,i}(t,f)\rangle \Vert }^{\frac{1}{2}}{\Vert \langle {W}_{j,j}(t,f)\rangle \Vert }^{\frac{1}{2}}}$ for $i,j=1,\ldots ,M$, where 〈·〉 denotes a smoothing operator both in time and frequency^39^.

In bivariate data analysis, the wavelet coherence is defined as ${{\rm{\Gamma }}}_{i,j}^{2}(t,f)=|{C}_{i,j}(t,f){|}^{2}$. To extend this idea to the general case of $M\geqslant 2$ signals, we can define a matrix ${\boldsymbol{\Sigma }}(t,f)$ at every point in the time-frequency domain containing all the pairwise coherence spectra:

1 $${\boldsymbol{\Sigma }}(t,f)=[\begin{array}{cccc}1 & {C}_{1,2}(t,f) & \ldots  & {C}_{1,M}(t,f)\\ {C}_{2,1}(t,f) & 1 & \ldots  & {C}_{2,M}(t,f)\\ \vdots  & \vdots  & \ddots  & \vdots \\ {C}_{M,1}(t,f) & {C}_{M,2}(t,f) & \ldots  & 1\end{array}],$$

The time-varying spatial coherence (TVSC) can be defined by

2 $${\rm{\Psi }}(t,f)=\frac{1}{M-1}({\lambda }_{{\rm{\max }}}^{{\rm{\Sigma }}}(t,f)-1),$$

where ${\lambda }_{{\rm{\max }}}^{{\rm{\Sigma }}}(t,f)$ denotes the largest eigenvalue of the spectral matrix ${\boldsymbol{\Sigma }}(t,f)$. In case of stationary observations, eigenvalues of the covariance matrix are commonly used in radio communications for detecting spatial correlations between time-invariant time series^40^.

The values of ${\rm{\Psi }}(t,f)$ are bounded between $0\leqslant {\rm{\Psi }}(t,f)\leqslant 1$, reaching the maximum when all the *M* signals are locally -in the time-frequency plane- pairwise correlated (${\boldsymbol{\Sigma }}(t,f)$ becomes an all-ones matrix with ${\lambda }_{{\rm{\max }}}^{{\rm{\Sigma }}}(t,f)=M$); and the minimum when all signals are completely uncorrelated (${\boldsymbol{\Sigma }}(t,f)={\bf{I}}$ and ${\lambda }_{{\rm{\max }}}^{{\rm{\Sigma }}}(t,f)=1$).

Interestingly, for the case $M=2$, ${\boldsymbol{\Sigma }}(t,f)$ is given by the matrix $$[\begin{array}{cc}1 & {C}_{1,2}(t,f)\\ {C}_{2,1}(t,f) & 1\end{array}]$$, whose largest eigenvalue is ${\lambda }_{{\rm{\max }}}^{{\rm{\Sigma }}}(t,f)=1+|{C}_{1,2}(t,f)|$, which yields ${\rm{\Psi }}(t,f)=({\lambda }_{{\rm{\max }}}^{{\rm{\Sigma }}}(t,f)-1)=|{C}_{1,2}(t,f)|$. In the bivariate case, this therefore reduces the TVSC to the classic definition of the wavelet coherence ${{\rm{\Psi }}}^{2}(t,f)={{\rm{\Gamma }}}^{2}(t,f)$.

### Detecting significant coherence

In wavelet-based analysis, test statistics are strongly affected by data’s structure, the mother wavelet’s properties, and by the smoothing applied^7–10,39^. In this work, the statistical properties of ${\rm{\Psi }}(t,f)$ under the null hypothesis *H*_0_ of *M* uncorrelated processes are determined by Monte Carlo simulation. To do this, we generate a number of surrogate data realisations ${\hat{{\bf{x}}}}^{j}(t),j=1,\ldots ,K$ by repeating the randomisation procedure *K* times. In our examples $K=100$ was used but the influence of the value of *K* has been also tested (see Supplementary Information). The statistical significance of ${\rm{\Psi }}(t,f)$ values was assessed by a z-test to quantify the statistical deviation from those values obtained in the ensemble of surrogate data. To correct for multiple testing, the false discovery rate (FDR) method was applied^41^. With this approach, the threshold of significance was set such that the expected fraction of false positives over the time-frequency plane is restricted to $q\leqslant 0.05$.

### Nonstationary surrogate data

A surrogate time series $\hat{{\bf{x}}}(t)$ can be obtained by randomising the phase structure of the original signal **x**(*t*) in the time-frequency domain. As the Morlet wavelet is a complex function, we can therefore write the wavelet transform ${W}_{x}(t,f)$ in terms of its phase ${\varphi }_{x}(t,f)={\tan }^{-1}\frac{\Im ({W}_{x}(t,f))}{\Re ({W}_{x}(t,f))}$ and modulus $|({W}_{x}(t,f))|$. The different steps of the wavelet-based surrogate algorithm are the following:

1. generate a Gaussian white noise time series to match the original data length,
2. derive the wavelet transform of this noise to extract the phase ${\varphi }_{{\rm{noise}}}(t,f)$,
3. combine this randomised phase and the WT modulus of the original signal to obtain a surrogate time-frequency distribution ${W}_{\hat{x}}(t,f)=|({W}_{x}(t,f))|\,\exp (i{\varphi }_{{\rm{noise}}}(t,f))$,
4. a nonstationary surrogate time series $\hat{{\bf{x}}}(t)$ is reconstructed by taking the real part of the inverse wavelet transform of ${W}_{\hat{x}}(t,f)$,
5. rescale the surrogate $\hat{{\bf{x}}}(t)$ to the distribution of the original time series by sorting the data (after a wavelet filtering in the frequency band of interest) according to the ranking of values of the wavelet-based surrogate^11^,

As its Fourier-based counterpart, our scheme can be iteratively repeated (by replacing the phase in step 2, by the phase of $\hat{{\bf{x}}}(t)$) to better adjust the time-frequency content of the surrogate data with that of the original time series (see Supplementary Information). Since Morlet wavelet transform is a bandpass filter, the inverse wavelet transform allows a reconstruction of the original time series by summing over a set of filtered waves^6,27^. Throughout the paper, the set of scales (frequencies) were selected such that the relative error in the wavelet reconstruction of the original time series was lower than 0.01.

### Datasets

To illustrate the detection of dynamic spatial correlation patterns on real-world time series, we study two systems: *i)* the weekly measles case notifications in seven large English cities studied in previous works^30,31^; and *ii)* an electroencephalographic (EEG) recording from a pediatric subject with intractable epileptic seizures^28,29^. Although our approach is applicable to any neuroimaging functional method (e.g. EEG, fMRI, and MEG signals) here we use the EEG as this modality of acquisition has the major feature that collective neural behaviors, i.e., synchronization of cortical assemblies are reflected as time-varying interactions between EEG signals. The file studied here contains 21 EEG signals sampled at 256 Hz according to the 10–20 bipolar montage^28^.

In this study, all time series are first centered and set to have zero mean and unit variance. The TVSC values are then computed and compared with the distribution of ${\rm{\Psi }}(t,f)$ under *H*_0_ obtained from surrogates. Throughout this work, the number of scales of the wavelet decomposition was selected such that an accurate reconstruction of the original signal was obtained, and the non-stationary oscillations and transient events observed in the time series were accurately captured.

### Models

We test the performance of our framework to detect spatial coherent components on two synthetic datasets with time-varying structure. In the first benchmark, the spatial system consists of 5 linear oscillators described by the following autoregressive (AR) model:

3 $$\begin{array}{rcl}{x}_{1t} & = & 0.95\sqrt{2}{x}_{1(t-1)}-0.9025{x}_{1(t-2)}+{\varepsilon }_{1t},\\ {x}_{2t} & = & 0.6{x}_{2(t-1)}-0.3{x}_{2(t-2)}+{k}_{2}{x}_{1(t-1)}+{\varepsilon }_{2t}\\ {x}_{3t} & = & 0.8{x}_{3(t-1)}-0.5{x}_{2(t-2)}+0.4{x}_{1(t-1)}+{\varepsilon }_{3t},\\ {x}_{4t} & = & {k}_{1}{x}_{1(t-2)}+0.25\sqrt{2}{x}_{4(t-1)}+0.25\sqrt{2}{x}_{5(t-1)}+{\varepsilon }_{4t},\\ {x}_{5t} & = & -0.25\sqrt{2}{x}_{4(t-1)}+0.25\sqrt{2}{x}_{5(t-1)}+{\varepsilon }_{5t}\end{array}$$

where *t* denotes a discrete time index, ${\varepsilon }_{i}$ are independent white noise processes with zero means and unit variances, and *k*_*i*_ are coupling strengths. Here, we set ${k}_{1}=0$ and ${k}_{2}=0.15$ for $t < 1000$; and ${k}_{1}=-\,0.5$ and ${k}_{2}=0.4$ for $t\geqslant 1000$.

Although the measure of time-varying spatial coherence is supposed to capture linear interactions, numerical evidence shows that ${\rm{\Psi }}(t,f)$ still provides a qualitative description in case of nonlinear oscillators. Indeed, we consider a network of $i=1,\ldots ,10$ coupled non-identical chaotic Rössler oscillators. The equations of motion read

4 $$\begin{array}{rcl}{\dot{x}}_{i} & = & -{\omega }_{i}{y}_{i}-{z}_{i}+\lambda [\sum _{j}\,\xi ij({x}_{j}-{x}_{i})]+{\sigma }_{i}{\eta }_{i},\\ {\dot{y}}_{i} & = & {\omega }_{i}{x}_{i}+0.165{y}_{i},\\ {\dot{z}}_{i} & = & 0.2+{z}_{i}({x}_{i}-10)\end{array}$$

where *λ*(*t*) is the time-varying coupling strength, $\xi ij$ are the elements of the coupling matrix (a random graph with an average number of links per node ${k}_{m}=4$); ${\omega }_{i}$ is the natural frequency of the *i*^th^ oscillator (randomly assigned from a uniform distribution with values between $0.98\leqslant {\omega }_{i}\leqslant 1.1$); ${\eta }_{i}$ denotes a Gaussian delta correlated noise with $\langle {\eta }_{i}(t)\rangle =0$ and $\langle {\eta }_{i}(t){\eta }_{i}(t^{\prime} )\rangle =2D\delta (t-t^{\prime} )$, $D=0.01$. Coupling strength *λ* varies with time as follows: $\lambda =0.5$ for $500 < t < 900$ and $\lambda =0.001$ elsewhere.

## Supplementary information

Supplementary Material

## Data Availability

In our study we have used previously released, freely available datasets. The measles dataset were obtained from the Prof. B. Bolker’s website at the McMaster University, Canada. They are freely available at https://ms.mcmaster.ca/~bolker/measdata.html. The EEG data was obtained from the open repository CHB-MIT Scalp EEG Database at https://www.physionet.org/pn6/chbmit. In case of any difficulty in obtaining the datasets mentioned above, the corresponding author can provide the data used upon request.

## Code Availability

Matlab and R codes implementing the non-stationary surrogate algorithm (with examples from the paper) are available at the open access repository https://zenodo.org (uploads 1213657 and 1213696).
