# Supplementary material for: Detecting dynamic spatial correlation patterns with generalized wavelet coherence and non-stationary surrogate data
Source: Sci Rep. 2019 May 14;9:7389. doi: 10.1038/s41598-019-43571-2 (PMC6517435; doi:10.1038/s41598-019-43571-2)
Supplement: Supplementary file 1 — Supplementary Material [file 41598_2019_43571_MOESM1_ESM.pdf]

SUPPLEMENTARY MATERIAL to "Detecting dynamic spatial correlation patterns with generalized wavelet coherence and non-stationary surrogate data"

M. Chavez[1] and B. Cazelles[2,3]

[1]CNRS UMR-7225, Hôpital de la Pitié-Salpêtrière. 75013 Paris, France
[2]IRD-UPMC UMI-209, UMMISCO, 93143 Bondy, France
[3]CNRS UMR-8197, IBENS, Ecole Normale Supérieure. 75005 Paris, France

Replication of the time-frequency structure with other surrogate algorithms. To compare the ability of our method to replicate the time-frequency (TF) distribution of the original data, we estimate the error:

$$e^2 = \frac{\| |W_{\widehat{x}}(t,f)| - |W_x(t,f)| \|_F}{\| |W_x(t,f)| \|_F} \tag{S-1}$$

where $\|A\|_F = \sqrt{\mathrm{Tr}(AA^T)}$ denotes the Frobenius norm of real matrix A, and $W_{\widehat{x}}(t,f)$ and $W_x(t,f)$ are the wavelet transforms of surrogate and original data, respectively.

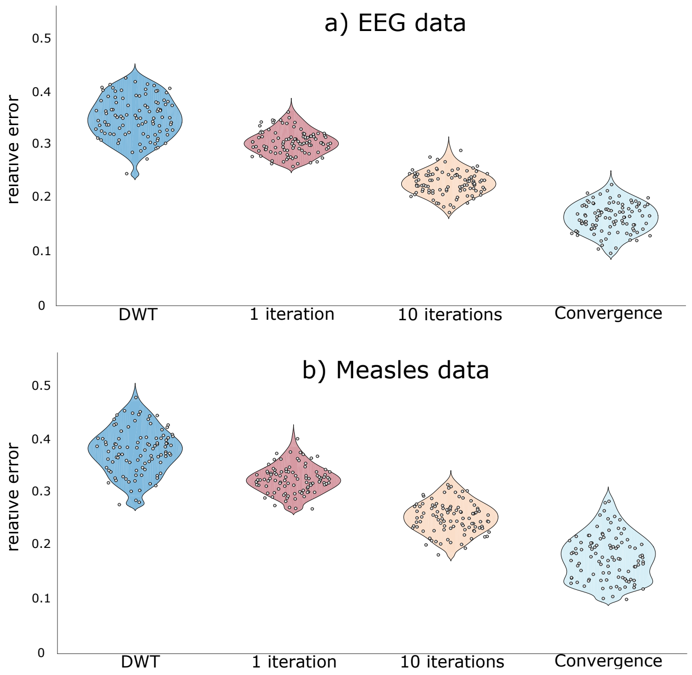

FIG. S-1. Replication of the TF distributions by different surrogate algorithms. Violin plots represent distributions of error values over 100 surrogates (gray points). The error of DWT-based algorithm is significantly larger than those obtained by our method at different number of iterations (a right-tailed Wilcoxon rank-sum test, $p \leqslant 0.05$).

To compare our method, different surrogating algorithms are applied to the two time series studied in Figs. 1 and 2 of the main text (the EEG and measles time series). We firstly compare our algorithm with a DWT-based method, i.e. the iAAWT algorithm (refer to Ref. [1] for detailed description of the algorithm). We also apply an improved iterative version of our method, in which an approximation of the original TF distribution is refined with each iteration until it is determined to be sufficiently accurate, at which time the procedure terminates.

In Fig. S-1 we report the normalized error produced by different surrogate replicates of original TF distributions. By construction, the histograms of the original data are exactly replicated by all the algorithms. For each time series, 100 surrogates are generated and the error computed. Our algorithm is applied in its simple version (a single iteration), after ten iterations, and finally for more than 30 iterations. Although the DWT-based algorithm replicates the multiscale structure of original data, our algorithm does a better job in this respect. Even after a single iteration, our algorithm reproduces better the non-stationary oscillations of studied time series. The error differences yielded by

our method is statistically smaller than that produced by the DWT-based method, as assessed by a non-parametric test (Wilcoxon-Mann-Whitney test, $p \leqslant 0.05$).

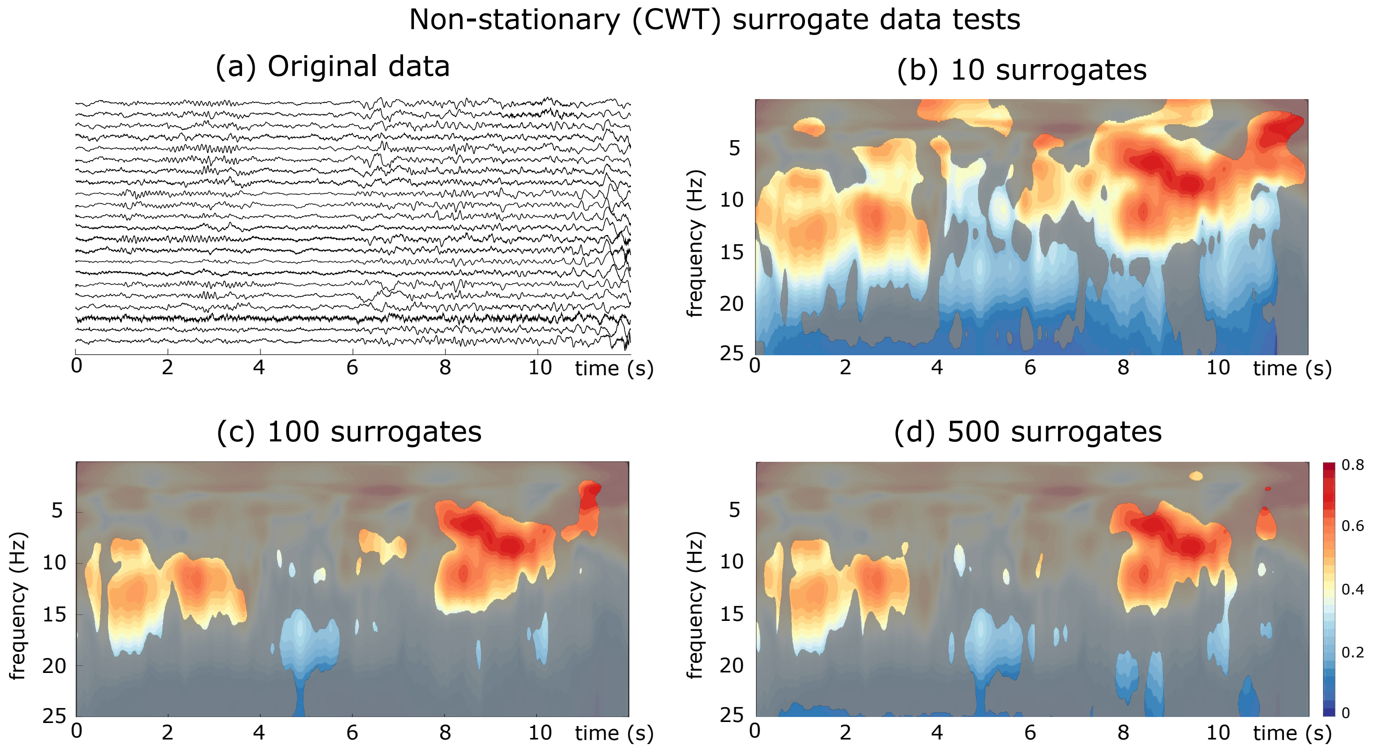

FIG. S-2. $\Psi(t, f)$ values estimated from epileptic EEG data and statistical significances obtained from different surrogate data tests. a) The original time series; surrogate data test obtained with the continuous wavelet transform (CWT) and b) $N = 10$, c) $N = 100$, and d) $N = 500$ realisations. Color maps code for $\Psi(t, f)$ values. Unmasked color regions in panels B-F indicate the significant levels. Black transparent maps indicate the cones of influence that delimit the time-frequency regions not influenced by edge effects.

Detection of coherent patterns with different number of surrogates. – We study more in detail the performances of our algorithm to detect non-stationary spatial coherent components. We evaluate the detection of significant coherent patterns for different number of surrogates generated by the simplest version of our algorithm (a single iteration) in the same real-world multivariate data studied in the main text: the epileptic EEG recordings and the measles dataset. Significant coherent patterns were detected as statistically different from those obtained from surrogates, by a Z-test corrected by a FDR at $q \leqslant 0.05$.

Results in Fig. S-2 clearly show that, for the EEG data, a test of significance based on a reduced number of of surrogates (10 realisations) yields to the detection of large and spurious synchronous regions, as those detected between $15 - 25$ Hz during practically the whole recording. In contrast, for 100 or 500 realisations, the detection based on our method considerably reduces the number of false coherent patches, and it clearly identifies the main regions with the highest spatial coherence, i.e. the characteristic short fast oscillatory behavior ($f \approx 15$ Hz) around $t = 3 - 4$ s just before the slow and large oscillations accompanying the epileptic seizure.

Results shown in Fig. S-3 indicate that, for the detection of coherent spatial patterns in measles data, tests based on a reduced number of non-stationary surrogate time series, practically no significant spatial coherent patterns are detected. In contrast , when the number of surrogate is increased (100 or 500) our approach clearly identifies the high spatial correlation between the major epidemic (mainly biennial) component of time series in the pre-vaccine era.

These supplementary results suggest that, when a sufficient number of surrogates is applied ($N \geqslant 100$), our test constitutes a good criterion to assess spatial coherence in the case of time series with time varying spectra.

[1] C. J. Keylock, Phys. Rev. E **95**, 032123 (2017).

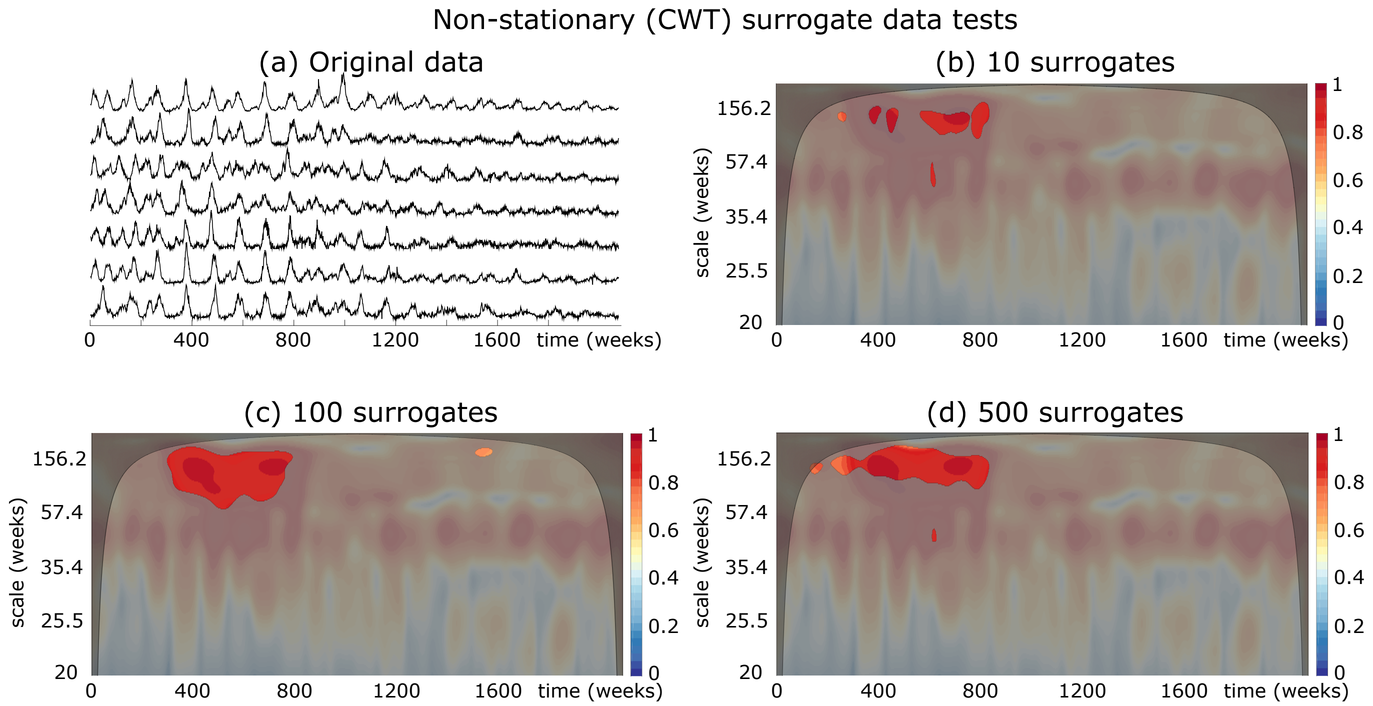

FIG. S-3. $\Psi(t, f)$ values estimated from measles data and statistical significances obtained from different surrogate data tests. Missing values in each original time series were imputed using a local average, i.e. the mean of the two neighboring time points. Same stipulations as in the caption of Figure S-2
